# MAGED2 Depletion Promotes Stress-Induced Autophagy by Impairing the cAMP/PKA Pathway

**DOI:** 10.3390/ijms241713433

**Published:** 2023-08-30

**Authors:** Sadiq Nasrah, Aline Radi, Johanna K. Daberkow, Helmut Hummler, Stefanie Weber, Elie Seaayfan, Martin Kömhoff

**Affiliations:** 1 Department of Pediatrics, University Hospital Giessen and Marburg, Philipps University Marburg, 35043 Marburg, Germany; sadiq.nasrah@uni-marburg.de (S.N.); radi@staff.uni-marburg.de (A.R.); hummler@staff.uni-marburg.de (H.H.); stefanie.weber@med.uni-marburg.de (S.W.); 2 Faculty of Medicine, Justus Liebig University Giessen, 35392 Giessen, Germany; johanna.k.daberkow@med.uni-giessen.de

**Keywords:** MAGED2, autophagy, cellular stress, cAMP, G-alpha-S, Bartter’s syndrome, hypoxia nephrogenesis

## Abstract

Melanoma-associated antigen D2 (MAGED2) plays an essential role in activating the cAMP/PKA pathway under hypoxic conditions, which is crucial for stimulating renal salt reabsorption and thus explaining the transient variant of Bartter’s syndrome. The cAMP/PKA pathway is also known to regulate autophagy, a lysosomal degradation process induced by cellular stress. Previous studies showed that two members of the melanoma-associated antigens MAGE-family inhibit autophagy. To explore the potential role of MAGED2 in stress-induced autophagy, specific MAGED2-siRNA were used in HEK293 cells under physical hypoxia and oxidative stress (cobalt chloride, hypoxia mimetic). Depletion of MAGED2 resulted in reduced p62 levels and upregulation of both the autophagy-related genes (ATG5 and ATG12) as well as the autophagosome marker LC3II compared to control siRNA. The increase in the autophagy markers in MAGED2-depleted cells was further confirmed by leupeptin-based assay which concurred with the highest LC3II accumulation. Likewise, under hypoxia, immunofluorescence in HEK293, HeLa and U2OS cell lines demonstrated a pronounced accumulation of LC3B puncta upon MAGED2 depletion. Moreover, LC3B puncta were absent in human fetal control kidneys but markedly expressed in a fetal kidney from a MAGED2-deficient subject. Induction of autophagy with both physical hypoxia and oxidative stress suggests a potentially general role of MAGED2 under stress conditions. Various other cellular stressors (brefeldin A, tunicamycin, 2-deoxy-D-glucose, and camptothecin) were analyzed, which all induced autophagy in the absence of MAGED2. Forskolin (FSK) inhibited, whereas GNAS Knockdown induced autophagy under hypoxia. In contrast to other MAGE proteins, MAGED2 has an inhibitory role on autophagy only under stress conditions. Hence, a prominent role of MAGED2 in the regulation of autophagy under stress conditions is evident, which may also contribute to impaired fetal renal salt reabsorption by promoting autophagy of salt-transporters in patients with MAGED2 mutation.

## 1. Introduction

Bartter’s syndrome (BS) is a rare inherited disease caused by mutations in the salt transporters of the thick ascending limb of the loop of Henle (TAL) and/or their regulatory subunits: *SLC12A1* encoding NKCC2 (BS 1), *KCNJ1* encoding ROMK (BS 2), *CLCNKB* encoding CLC-Kb (BS 3), *BSND* encoding Barttin (BS 4a), and *CLCNKA* and *CLCNKB* encoding CLC-Ka, CLC-Kb, respectively (BS 4b) and *MAGED2* encoding MAGED2 (BS 5) [1]. BS is characterized by polyuria, hypokalemia, hypercalciuria, alkalosis, nephrocalcinosis and low to normal blood pressure. Aside from the majority of cases of BS3, all other types of BS present with polyhydramnios, due to the limited capacity of the placenta to reabsorb electrolytes [2]. In contrast to Bartter’s syndromes 1–4, Bartter’s syndrome 5 resolves spontaneously despite its most severe initial presentation [3,4].

Our research provides an explanation for the transient character of Bartter’s syndrome 5 by showing that under hypoxic conditions, MAGED2 functions as a master regulator of the cAMP/PKA pathway by regulating the endocytosis of Gαs through MDM2-dependent ubiquitination [5,6], which in turn is required for renal salt reabsorption by NKCC2 and NCC [7,8]. Hence, the spontaneous resolution of transient Bartter’s syndrome caused by *MAGED2* mutations may result from a developmental increase in oxygen supply to the kidney, which renders MAGED2 dispensable as Gαs can function independently under normoxia [1]. MAGED2 is a member of the melanoma-associated antigens (MAGE) gene family, which is evolutionarily conserved in eukaryotes [9] and is considered the ancestral member of the entire family of approximately 40 members in humans [10]. The human MAGE gene family is divided into two subfamilies: Type I MAGEs (MAGEs A–C), which are mainly expressed in testes and cancer, and Type II MAGEs (MAGEs D–G), which are expressed ubiquitously. MAGE proteins regulate the activity of E3 ubiquitin ligases [11] through the MAGE homology domain (MHD) [12]. There are >700 different E3 ligases, which affect client proteins through non-degradative ubiquitination (such as protein trafficking) or degradative (“proteasomal”) ubiquitination [13]. Apart from that, MAGE proteins also exhibit additional biochemical functions that are independent of E3 ubiquitin ligases [9], and ongoing research is discovering new functions of these proteins. The role of MAGED2 in the protection against hypoxic stress fits well with previous reports showing diverse members of the MAGE-gene family protecting against diverse stressors. For example, Mage-A2/A6/A8 knockout (KO) mice have compromised spermatogenesis following genotoxic or nutritional stress [14]. Moreover, expression of MAGEA3/6 is restricted to germline cells but is re-expressed in tumor cells following demethylation of the promoter, where it promotes proteasomal degradation of AMPK via the E3 ubiquitin ligases TRIM28. The latter inhibits AMPK-dependent autophagy, enhances proliferation downstream of mTORC1, and thereby promotes tumorigenicity [15].

Based on our previous finding that MAGED2 protects against hypoxic stress [5], a known inducer of autophagy, the effects of MAGED2 depletion on the induction of autophagy under different stressors in vitro were analyzed. We showed that MAGED2 blocks autophagy in three different cell lines exposed to physical and chemical hypoxia. Importantly, the markedly increased expression of LC3B in a fetal kidney from a patient with truncating mutations in MAGED2 but not in two corresponding age-matched control kidneys confirms the relevance of our in vitro findings under hypoxia. Because chemical hypoxia stresses cells by different pathways compared to physical hypoxia, we tested endoplasmic-reticulum-, nutritional- and genotoxic stressors, which all induced autophagy in MAGED2 depleted cells. Finally, we revealed that under hypoxia, MAGED2 depletion promotes autophagy by inhibiting the cAMP/PKA pathway. This effect was reversed by forskolin (a cAMP/PKA pathway activator), in contrast to GNAS knockdown (cAMP/PKA pathway inhibition), which promoted it. 

## 2. Results

### 2.1. Induction of Autophagy upon MAGED2 Depletion under Hypoxia

**Figure 1:**
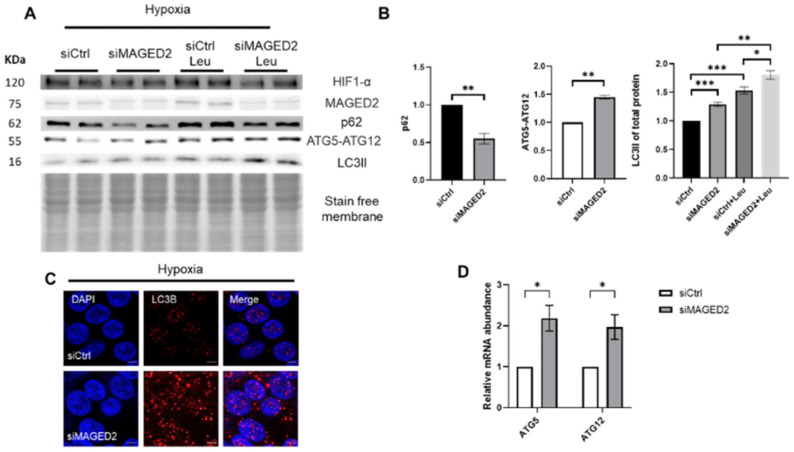
MAGED2 depletion induces autophagy under hypoxic conditions. HEK293 cells were transfected with control or MAGED2 siRNAs. Upon confluency 24–48 h post transfection, cells were exposed to physical hypoxia overnight. Total cell lysates were separated by SDS-PAGE and blotted for p62, ATGs and LC3B detection. Blotting for HIF-1α- was carried out to confirm its induction under hypoxia. Immunocytochemistry was carried out in parallel in HEK293 cells transfected and stressed similarly prior to incubation with LC3B antibody. (**A**) Representative Western blot images demonstrate decreased p62 levels, elevated ATG5-ATG12 complex levels and a higher LC3II abundance upon MAGED2 depletion in the presence of physical hypoxia. Leupeptin assay confirmed the induced autophagy where the highest LC3II accumulation corresponded to cells where MAGED2 is knocked down. (**B**) Densitometric analysis of p62, ATG5-ATG12 conjugate and LC3II from the immunoblot A. All samples shown on individual blots are from the same experiment and each blot represents an example of three independent experiments. (**C**) Representative immunofluorescence images displaying LC3B staining in control and MAGED2-transfected HEK293 cells under physical hypoxia. Scale bar 5 µm. (**D**) This notion was further supported by qRT-PCR, where the quantity of ATG5 and ATG12 was determined in HEK293 cells transfected with control or MAGED2 siRNAs and exposed to hypoxia. Total mRNA was isolated, and the relative mRNA amounts of both genes were measured. Statistical significance was determined by unpaired Student’s *t*-test. Bar graphs show mean ± SEM, * *p* ≤ 0.05, ** *p* ≤ 0.01, *** *p* ≤ 0.001.

To investigate the regulatory role of MAGED2 on autophagy under hypoxic stress, HEK293 cells were transfected with control or MAGED2 siRNAs and exposed at confluency to normoxia or physical hypoxia (1% O_2_, 5% CO_2_, 94% N_2_) overnight. Immunoblotting confirmed induction of HIF-1α under hypoxia. Under normoxia, the depletion of MAGED2 had no effect on ATG5-ATG12 conjugate levels, which alongside ATG16 are essential for autophagosome formation, nor on LC3II abundance. In contrast, physical hypoxia induced the expression of ATG5-ATG12 conjugates and led to a statistically significantly increased LC3II abundance in MAGED2-depleted cells, as shown by immunoblotting (Figure S1 and Figure 1A,B). The autophagy flux was further assayed using p62 and leupeptin, the protease inhibitor which blocks autophagy substrate degradation and allows monitoring LC3B turnover. Indeed, knocking down MAGED2 in HEK293 cells concurred with reduced p62 levels and the highest LC3II accumulation when treating with leupeptin (Figure 1A,B). The measurement of ATG5 and ATG12 mRNA level by RT-qPCR showed an increase in its expression when MAGED2 was depleted (Figure 1D). To independently confirm this observation, accumulation of LC3B puncta was analyzed by immunocytochemistry as demonstrated in Figure 1C; the abundance of puncta was markedly elevated in MAGED2-depleted cells.

Induction of autophagy upon MAGED2 depletion in response to hypoxic stress was validated in HeLa (Figure S2A,B) and U2OS (Figure S2C,D) cells (U2OS harbors endogenous wild-type p53 in contrast to HeLa and HEK293 cells), thereby excluding the possibility that induction of autophagy in HeLa and HEK293 cells results from a lack of p53, which could induce or inhibit autophagy by regulating the AMPK/mTOR pathway. A significantly induced autophagic response as judged by immunoblotting and immunocytochemistry upon MAGED2 depletion was detected, thus validating the potential inhibitory role of MAGED2 on autophagy independently of p53.

**Figure 2:**
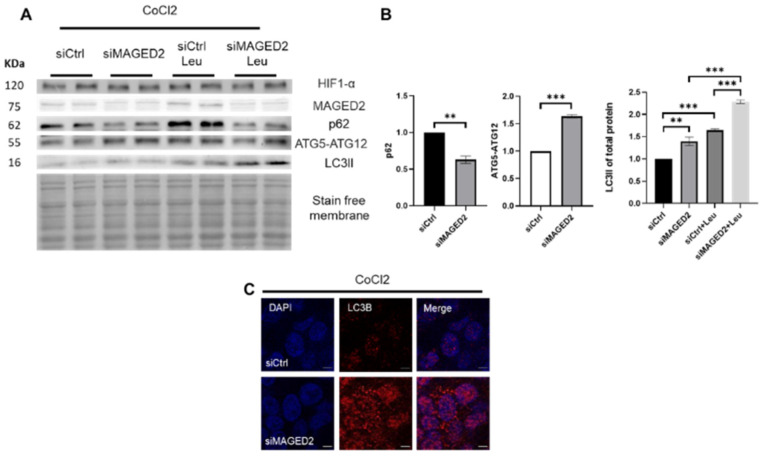
Cobalt chloride induces autophagy in MAGED2-depleted HEK293 cells. HEK293 cells were transfected with control or MAGED2 siRNAs. Upon confluency 24–48 h post transfection, cells were treated with cobalt chloride (“chemical hypoxia”, CoCl_2_, 300 µM) for 14–16 h. Total cell lysates were separated by SDS-PAGE and blotted for p62, ATGs and LC3B detection. Of note, HIF-1α immunoblotting confirmed the hypoxic condition. Immunocytochemistry was carried out in parallel in HEK293 cells transfected and stressed similarly prior to incubation with LC3B antibody. (**A**) Representative Western blot images from HEK293 cells demonstrate reduced p62 levels, increased ATG5-ATG12 conjugate levels and a higher LC3II prevalence upon MAGED2 depletion. Coincubation with leupeptin (100 µM) led to an increased LC3II accumulation, which confirmed induction of autophagy. (**B**) Densitometric analysis of p62, ATG5-ATG12 conjugate and LC3II from immunoblot A. All samples shown on individual blots are from the same experiment, and each blot represents an example of three independent experiments. Bar graphs show mean ± SEM, ** *p* ≤ 0.01, *** *p* ≤ 0.001. (**C**) Representative immunofluorescence images displaying LC3B staining in control and MAGED2-transfected HEK293 cells treated with CoCl_2_. Scale bar 5 μM.

Similar to physical hypoxia, cobalt chloride, an oxidative stress inducer, significantly induced autophagy (Figure 2A,B) and led to marked abundance of LC3B puncta (Figure 2C, Figures S3A and S4A) as judged by immunocytochemistry upon MAGED2 depletion. These findings show the important role that MAGED2 plays in inhibiting autophagy under oxidative stress conditions.

### 2.2. Increased Abundance of LC3B in a Fetal Kidney from a Patient with a Truncating Mutation in MAGED2

**Figure 3:**
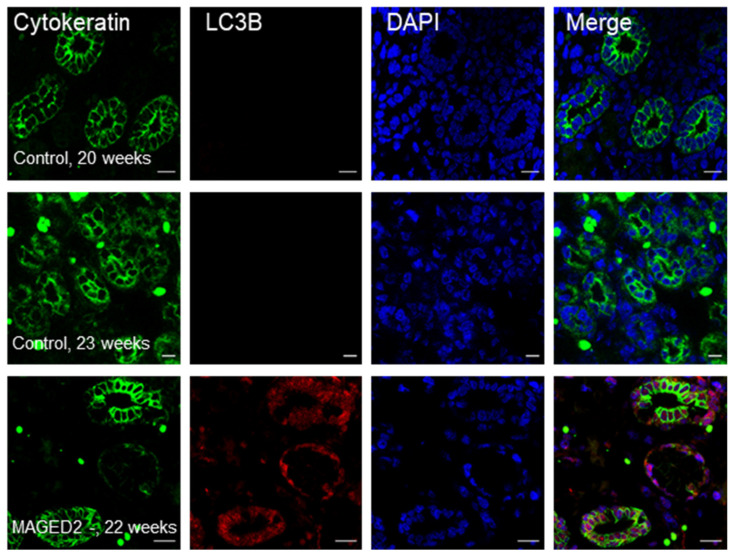
Enhanced expression of LC3B in the kidney from a fetus with transient Bartter’s syndrome compared to age-matched controls. Representative immunostaining of LC3B in fetal kidney sections from aborted cases. Staining was performed using LC3B and cytokeratin antibodies visualized Alexa 555, red, and Alexa 488, green, respectively. Scale bar 20 μM.

To independently validate the relevance of our findings of enhanced autophagy in MAGED2-depleted hypoxic cells in vitro, the expression of LC3B was analyzed ex vivo in fetal kidneys from a developmental stage, which is characterized by abundant expression of the hypoxia-marker HIF-1α (hypoxia inducible factor 1α) [16]. In two individual fetal control kidneys (at 20 and 23 weeks of gestation, respectively) LC3B immunoreactivity was barely noticeable. In contrast, in a fetal kidney from a patient who died in utero at 22 weeks of gestation with transient Bartter’s syndrome caused by a truncating mutation (c.1038C→G; p.Y346*), LC3B immunoreactivity was abundantly detected primarily in the epithelia of medullary collecting ducts (co-labeled with cytokeratin) and to a lesser extent in the surrounding interstitial cells (Figure 3). 

### 2.3. ER-Stressors Induce Autophagy upon MAGED2 Depletion

The fact that both physical hypoxia and cobalt chloride, which stress cells very differently but both cause endoplasmic reticulum stress, induce autophagy suggests that other cellular stressors may also induce autophagy in MAGED2-depleted cells. Hence, we studied the effects of two endoplasmic reticulum stress inducers, namely tunicamycin, which disturbs the secretory pathway by inhibiting N-linked glycosylation, and brefeldin A (BFA), which blocks the exit of secretory proteins from the ER, thereby disintegrating the Golgi-apparatus [17].

**Figure 4:**
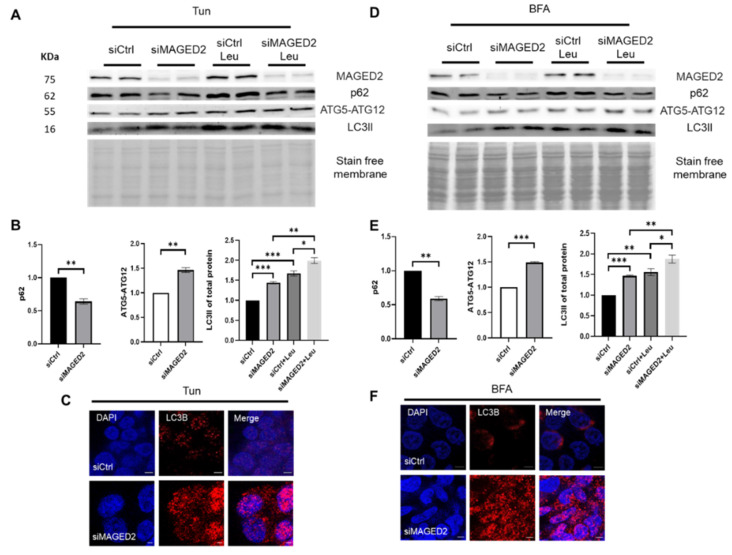
Autophagy is significantly induced by classical ER-stressors in MAGED2-depleted cells. Control or MAGED2 siRNAs were transfected into HEK293 cells. At 24–48 h post-transfection, cells were treated with the ER stressors 600 nM tunicamycin (Tun) overnight or 10 μM brefeldin A (BFA) for 2 h. SDS-PAGE was used to separate total cell lysates before blotting for p62, LC3B and ATGs detection. Immunocytochemistry was conducted in parallel, and HEK293 cells were stained for the accumulation of LC3B puncta after being transfected and treated with ER stressors. Representative Western blot images from HEK293 cells treated with tunicamycin (**A**) or BFA (**D**) show reduced p62 levels combined with increased levels of ATG5-ATG12 complex and higher LC3II abundance in MAGED2-depleted cells. Leupeptin treatment led to the highest LC3II accumulation because of blocked autophagic flux. (**B**,**E**) Densitometric analysis of p62, ATG5-ATG12 conjugate and LC3II of the immunoblots (**A**,**D**) respectively. All blots are from the same experiment, and each represents an example of three independent experiments. Bar graphs show mean ± SEM, * *p* ≤ 0.05, ** *p* ≤ 0.01, *** *p* ≤ 0.001. (**C**,**F**) LC3B staining in control and MAGED2-transfected HEK 293 cells treated with tunicamycin or BFA, respectively. The scale bar is 5 μM.

HEK293 cells were transfected with control or MAGED2 siRNA and treated with tunicamycin overnight. Immunoblotting showed a significantly higher LC3II abundance and ATG5-ATG12 complex expression in MAGED2-depleted cells along with reduced p62 levels (Figure 4A,B). Immunocytochemistry for LC3B puncta confirmed that autophagy was remarkably higher in MAGED2-depleted cells compared to control cells (Figure 4C, Figures S3B and S4B).

Similarly, HEK293 cells were treated with BFA for 2 h. Western blotting and immunocytochemistry experiments showed that MAGED2 depletion promotes autophagy in the presence of BFA (Figure 4D–F, Figures S3C and S4B). These findings support the idea that ER stress promotes autophagy in the absence of MAGED2.

### 2.4. Genotoxic Stress Promotes Autophagy in MAGED2 Depleted Cells

**Figure 5:**
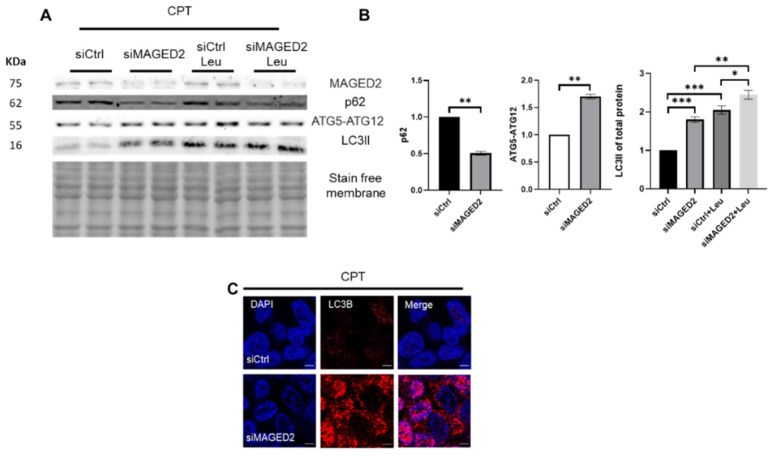
Genotoxic stress enhances autophagy in MAGED2-depleted cells. HEK293 cells were transfected with control or MAGED2 siRNAs. Cells were treated 24–48 h post-transfection with 10 µM camptothecin (CPT) overnight. Total cell proteins were separated using SDS-PAGE and then immunoblotted for p62, ATGs and LC3B detection. In parallel, immunocytochemistry for HEK293 cells was carried out to stain for LC3B puncta following the same protocol. (**A**) Representative Western blot images from HEK293 cells treated with CPT show decreased p62 expression, increased levels of ATG5-ATG12 complex and higher LC3II abundance upon MAGED2 depletion. Treatment with leupeptin blocked the autophagic flux and resulted in the highest LC3II accumulation when MAGED2 was depleted. (**B**) Densitometric analysis of p62, ATG5-ATG12 complex and LC3II from the immunoblots in (**A**). All blots are from the same experiment, and each represents an example of three independent experiments. Bar graphs show mean ± SEM, * *p* ≤ 0.05, ** *p* ≤ 0.01, *** *p* ≤ 0.001. (**C**) Representative immunofluorescence images showing LC3B staining in control and MAGED2-transfected HEK293 cells upon CPT treatment. Scale bar 5 µM.

Camptothecin (CPT) stabilizes the topoisomerase I cleavage complex [18] and induces cellular stress beyond DNA damage. It was shown that it elevates ER stress in various cancer cell lines [19,20] and enhances G_2_/M phase cell cycle arrest mediated by reactive oxygen species (ROS) [21]. The aim was to study its effects on autophagy in MAGED2-depleted cells. Similar to the other stressors, HEK293 cells showed higher induction of autophagy upon CPT treatment, as evidenced by significantly decreased p62 in conjugation with elevated LC3II and ATG5-ATG12 complex levels (Figure 5A,B) and markedly more LC3B puncta (Figure 5C) in MAGED2-depleted cells. Similar effects were observed in HeLa (Figure S3D) and U2OS (Figure S4D) cells.

### 2.5. Nutritional Stress Promotes Autophagy in MAGED2-Depleted Cells

**Figure 6:**
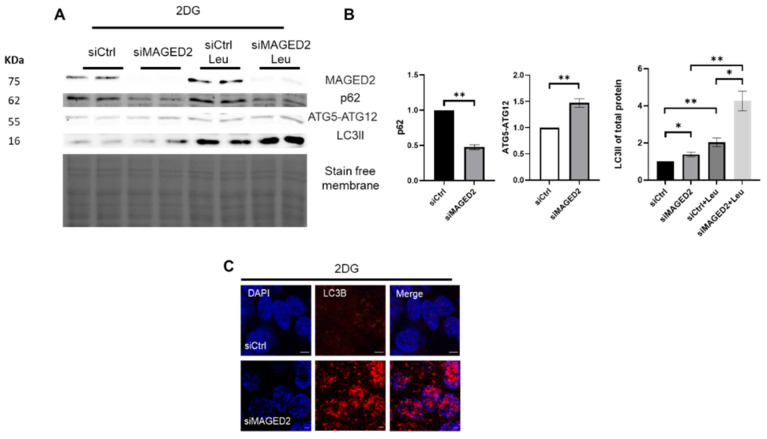
Nutritional stress promotes autophagy in MAGED2-depleted cells. Control or MAGED2 siRNAs were transfected into HEK293 cells. Cells were treated 24–48 h post transfection with 4 mM 2-Deoxy-D-glucose (2DG) for 30 min. SDS-PAGE was used to separate total cell lysates, which were next blotted for p62, ATGs and LC3B detection. Immunocytochemistry was conducted as mentioned before, and both HeLa and HEK293 cells were stained for the accumulation of LC3B puncta. (**A**) Representative Western blot images from HEK293 cells treated with 2DG shows decreased p62 levels, elevated expression of ATG5-ATG12 complex, higher LC3II abundance upon MAGED2 depletion and the highest ratio when co-incubating with Leupeptin. (**B**) Densitometric analysis of P62, ATGs and LC3II from the immunoblots in (**A**). All blots are from the same experiment, and each represents an example of three independent experiments. Bar graphs show mean ± SEM, * *p* ≤ 0.05, ** *p* ≤ 0.01. (**C**) Representative immunofluorescence images showing LC3B staining in control and MAGED2-transfected HEK293 cells upon 2DG treatment. Scale bar 5 µM.

2-Deoxy-D-glucose (2-DG) is a glucose analog, which induces nutritional stress by inhibiting glycolysis [22,23], thereby reducing cellular ATP. Due to its structural similarity with mannose, it also interferes with oligosaccharide synthesis, causing abnormal N-linked glycosylation and ER stress [24]. HEK293 cells were treated for 30 min with 2-DG. Immunoblotting and immunocytochemistry were performed to monitor the autophagy flux (Figure 6A,B), and the accumulation of LC3B puncta (Figure 6C), respectively, which both were increased in MAGED2 depleted cells. Increased accumulation of LC3B puncta was observed in HeLa (Figure S3E) and U2OS (Figure S4E) cells upon knockdown of MAGED2.

### 2.6. Activation of cAMP/PKA Pathway Reversed the Stress-Induced Autophagic Machinery upon MAGE2 Depletion 

**Figure 7:**
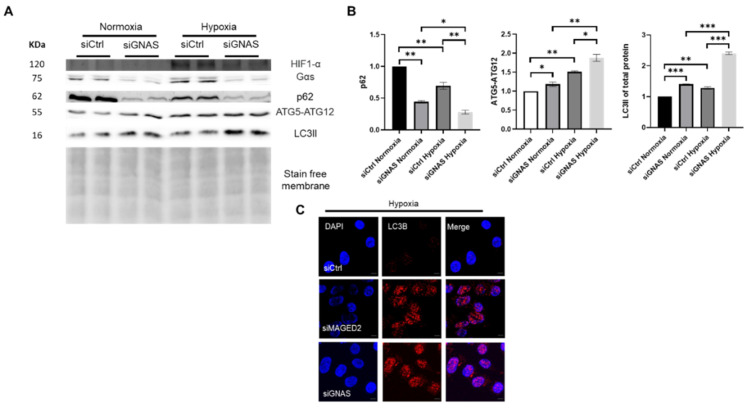
GNAS depletion induces autophagy under hypoxic conditions. Control or GNAS siRNAs were transfected into HEK293 cells. Upon confluency, 24–48 h post transfection, hypoxic stress was applied overnight for one set in a modular chamber while the other set was kept in normoxic conditions. Cells were then lysed and blotted for detection of p62, LC3B and autophagy-related genes. HIF-1α immunoblot confirmed hypoxia. (**A**) A representative Western blot from HEK293 cells demonstrates that GNAS depletion induced autophagy significantly under hypoxia where the low p62 levels and ATGs upregulation were accompanied by a higher conversion to the lipidated form LC3II under hypoxia. (**B**) Densitometric analysis of p62, ATG5-ATG12 complex and the LC3II from the immunoblot A. All samples shown on individual blots are from the same experiment, and each blot represents an example of three independent experiments. Bar graphs show mean ± SEM, * *p* ≤ 0.05, ** *p* ≤ 0.01, *** *p* ≤ 0.001. (**C**) Immunocytochemistry for HEK293 cells transfected with either control, MAGED2 or GNAS siRNAs and exposed to physical hypoxia for 14–16 h show the accumulation of LC3B puncta. Similar to MAGED2-depletion, knockdown of GNAS also led to an increased abundance of LC3B positive puncta. Scale bar is 5 µM.

We recently demonstrated that MAGED2 is essential under hypoxic conditions for correct localization of Gαs at the plasma membrane, cAMP generation and PKA activation [6]. Therefore, we asked whether induction of autophagy in our experiments resulted from impaired functioning of Gαs. Hence, we examined the effect of Gαs-depletion in HEK293 cells exposed to normoxia or physical hypoxia. Induction of HIF-1α protein confirmed hypoxia. Interestingly, knockdown of Gαs induced autophagy under both conditions, although it was much more pronounced under hypoxic stress (lowest p62 level combined with increased LC3II and ATG5-ATG12 levels) (Figure 7A,B). It also led to increased abundance of LC3B-positive puncta as assessed by immunocytochemistry (Figure 7C). Gαs depletion and the corresponding autophagic induction indicates cAMP/PKA implication. 

**Figure 8:**
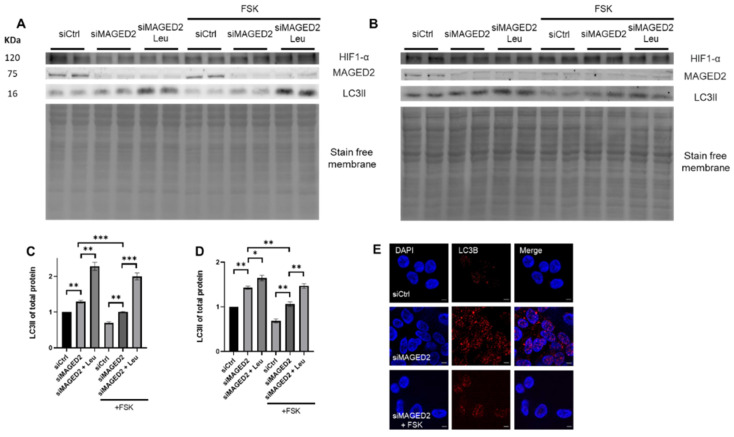
Forskolin reverses the induction of autophagy under stress conditions upon MAGED2 depletion. HeLa and HEK293 cells were both transfected with control and MAGED2 siRNA. After 24 to 48 h upon confluency, the media was changed to DMEM as control or DMEM containing 10 µM FSK before subjecting all cells to physical hypoxia overnight. SDS-PAGE was used to separate total cell proteins, which were further immunoblotted for LC3B detection. Hypoxic condition was verified by blotting for HIF-1α. Moreover, immunocytochemistry for HEK293 cells transfected with either control or MAGED2 siRNA and exposed upon confluency to overnight physical hypoxia with or without the addition of 10 µM of FSK was performed and the accumulation of LC3B puncta was analyzed. (**A**,**B**) Representative Western blot images from (**A**) Hela and (**B**) HEK293 cells treated with 10 µM FSK show a reduction in LC3B expression and a diminished autophagy induction upon FSK treatment. The promoted autophagy seen when MAGED2 is knocked-down is rendered to control levels by FSK addition. (**C**,**D**) Densitometric analysis of LC3B immunoblots in (**A**,**B**), respectively. All blots are from the same experiment, and each represents an example of three independent experiments. Bar graphs show mean ± SEM, * *p* ≤ 0.05, ** *p* ≤ 0.01, *** *p* ≤ 0.001. (**E**) Immunocytochemistry confirms that FSK addition to HEK293 cells prior to hypoxic stress reversed the observed autophagic induction, and the accumulation of puncta was rendered to control levels. Scale bar is 5 µM.

To independently confirm an inhibitory role of cyclic AMP elevating agents on autophagy in MAGED2 hypoxic cells, HeLa and HEK293 cells were transfected with control or MAGED2 siRNA, treated with FSK and exposed to physical hypoxia. Interestingly, immunoblot analysis revealed a reduction in LC3II levels and a diminished autophagic response upon forskolin treatment (Figure 8A–D). FSK treatment was confirmed to activate PKA in HeLa and HEK293 cells by immunoblotting against phosphorylated PKA substrates (Figure S5). This finding was supported by immunocytochemistry, which showed that the accumulation of LC3B puncta was abrogated when MAGED2-depleted, hypoxic cells were pre-exposed to FSK (Figure 8E). A similar effect was observed in MAGED2-depleted cells treated with tunicamycin and FSK (Figure S6).

## 3. Discussion

**Figure 9:**
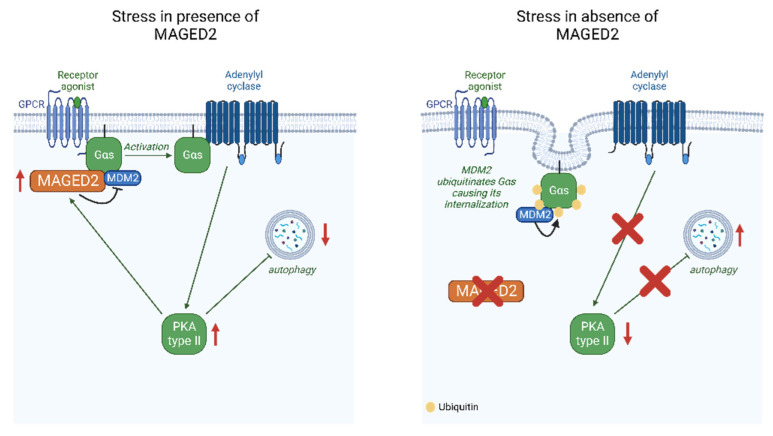
Proposed model for the role of MAGED2 under stress conditions (created with BioRender.com). Under stress, MAGED2 inhibits MDM2-dependent ubiquitination and endocytosis of Gαs. This ensures activation of the adenylate cyclase and cAMP generation and activation of PKA under stress. Reduced PKA activity impairs regulation of autophagy mediated by the cAMP/PKA pathway.

In this study, we demonstrated that MAGED2 blocks the induction of autophagy in three cell lines exposed to various stressors but not in unstressed cells. The in vitro data are independently supported by showing markedly enhanced abundance of LC3B puncta in the oxygen-deprived human fetal renal medulla of a patient carrying a mutation in MAGED2 but not in human fetal renal medulla from corresponding stages of renal development without a history of polyhydramnios. We also provide evidence that the inhibition of autophagy by MAGED2 requires activation of the cAMP/PKA pathway and does not rely on the functionality of p53 (Figure 9). 

Activation of the cAMP/PKA pathway is known to either activate or inhibit autophagy. In yeast, high glucose levels promote cAMP production, which inhibits autophagy by phosphorylation of ATG1 and ATG9, two key activators of macroautophagy [25]. In mammalian cells, cAMP can either inhibit [26,27,28] or promote [27,29] autophagy, depending on the cell type. Grisan and colleagues showed that the differential effects of cAMP on the regulation of autophagy depend on cell-type specific compartmentalization of PKA activity [30]. Of note, it was shown that only displacement of PKA type II (but not of PKA type I) reversed the effect of cAMP on autophagy, which concurs with our previous finding that MAGED2 regulates PKA type II [6]. In agreement with our data, knockdown of Gαs has been shown to promote autophagy in HeLa cells [31]. 

Concurring with the general notion that MAGE protein family members are protective against various forms of stress [9], we have previously shown that MAGED2 is required for a functional Gαs/cAMP/PKA pathway under hypoxic conditions [5,6]. Hence, our new finding that MAGED2 inhibits autophagy only under stressed conditions fits well with an important role of MAGED2, which is restricted to stress conditions. In this respect, our findings with MAGED2 differ from previous studies showing induction of autophagy upon deletion of MAGEA3/A6 in unstressed HeLa and U2OS cells [15], or unstressed MAGED1-depleted A549 and H1299 cells [32]. Given that activation of the cAMP/PKA pathway by forskolin abrogated autophagy in cells exposed to either physical hypoxia or the ER-stressor tunicamycin indicates that the latter stressor (like hypoxia) may also promote endocytosis of Gαs. Hence, stress-induced internalization of Gαs resulting from endocytosis induced by MDM2-dependent ubiquitination in the absence of MAGED2 [6] could be the switch activated by all stressors used in this study, which would explain why autophagy occurs only in MAGED2-depleted cells under stress in contrast to previous studies in MAGEA3/A6- or MAGED1-depleted cells. Analogously to the oncogenic potential of MAGEA3/A6 expression in tumors resulting in enhanced proliferation by limiting mTOR-dependent autophagy, a similar role of MAGED2 is conceivable, which is abundantly expressed in many types of cancers [33,34,35,36]. 

Previously, autophagy has been deemed dispensable for kidney development [37,38] in contrast to adult kidneys, where autophagy in diverse cells including glomerular mesangial and endothelial cells, podocytes and proximal tubule epithelial cells has been shown to be essential for maintaining kidney integrity and normal physiology [39]. Our study now points to a potential role of autophagy in developing nephrons as shown by abundant LC3B positivity in collecting ducts. At the moment, direct evidence that salt-transporters such as NCC and NKCC2, which we have already shown to require MAGED2 and are aberrantly expressed in transient Bartter’s syndrome [3], might be regulated by MAGED2 via autophagy, is missing. Of note, NCC has been shown to be degraded in lysosomes [40], which opens the possibility of regulation by autophagy. MAGED2 is abundantly expressed in the distal tubule in adult humans and rats starting from the cortical thick ascending limb of the loop of Henle down to the inner medullary collecting duct [3,41], where a number of cAMP-regulated salt-transporters are expressed [7]. Previously, induction of MAGED2 in the distal tubule in folic-acid induced acute kidney injury has been reported [42], in keeping with a role of MAGED2 in the protection against stress by preserving renal salt reabsorption in AKI. Of interest, rapamycin, which promotes autophagy by inhibiting mTOR [43], has been shown to alleviate hypertension in Dahl salt-sensitive rats [44], and deletion of raptor, which functions downstream of mTOR and inhibits autophagy, causes a Bartter’s syndrome/furosemide-like renal phenotype including massive polyuria and hypercalciuria [45].

One limitation of this study arises from the limited access to human fetal kidney tissues. In addition to the marked LC3B staining, quantitative (qPCR) or semiquantitative (Western blot) assays from fetal tissues would have been advantageous. The second limitation concerns the potential effects of MAGED2 on the protection of renal salt transporters, particularly NKCC2 and NCC, from hypoxia-mediated autophagy. This needs to be determined in detail in forthcoming studies.

In sum, our study reveals that MAGED2 inhibits autophagy via the cAMP/PKA pathway in cells exposed to a diverse set of stressors in vitro and ex vivo as shown by massive autophagy in collecting ducts of a MAGED2-deficient human fetal kidney. Our data may indicate a probable reoccurrence of renal salt wasting, upon exposure of MAGED2-deficient individuals to certain stressors.

## 4. Material and Methods

### 4.1. Cell Culture

DMEM Glutmax containing 10% fetal bovine serum superior (Sigma-Aldrich, Schnelldorf, Germany), penicillin (100 units/mL) and streptomycin (100 units/mL) was used to grow Human Embryonic Kidney (HEK293), Human Bone Osteosarcoma Epithelial Cells (U2OS Line) and HeLa cells (Table 1) at 37 °C in a humidified atmosphere of 5% CO_2_. Control and experimental cells were always derived and studied from the same flask and passage on the same day.

### 4.2. siRNA (Small Interfering RNA) Knockdown

The ON-TARGETplus SMARTpools of the siRNAs for control, MAGED2 and GNAS were purchased from Dharmacon (D-001810-10-05 and L-010825-00-0005). By reverse transfection according to the manufacturer’s instructions, cells were transfected with either control or MAGED2 siRNAs using DhramaFECT4.

### 4.3. Establishment of Cellular Stress

At 24–48 h post transfection, the medium of the confluent cells was changed to a media containing one of the chemical stressors; endoplasmic reticulum (ER) stressors, Tunicamycin (Tun, 600 nM) or Brefeldin A (BFA, 10 µM); a genotoxic stressor, Camptothecin (CPT, 10 µM); a nutritional stressor, 2-deoxy-D-glucose (2DG, 4 mM); or a hypoxia mimetic, Cobalt Chloride (CoCl_2_, 300 µM) which induces HIF1-α expression “Chemical Hypoxia”. Overnight incubation of cells in a standard humidifier at 37 °C was needed for Tun, CPT and CoCl_2_, while incubation with BFA and 2DG lasted only for 2 h and 30 min, respectively, to evade toxic effects. Physical hypoxia was performed overnight in a modular hypoxia incubator chamber (Billups-Rothenberg, Inc., San Diego, CA, USA, Cat. MIC-101), as described previously [5]. 

### 4.4. Immunocytochemistry

Cells were cultured in poly-L-lysine coated chamber slides. Fixation of cells was done at 4 °C by using 4% paraformaldehyde in PBS for 20 min followed by permeabilization in 0.1% Triton X-100 for 5 min at 4 °C. Cells were incubated afterwards with DAKO (antibody diluent with background-reducing components) for 30 min to prevent nonspecific antibody binding. Rabbit LC3B antibody (1:2000) was used as a primary antibody for 1 h at room temperature, and the Alexa Fluor Plus 555-coupled goat anti-Rabbit IgG antibody (1:500) was used as a secondary antibody. Visualization was done after mounting with VECTASHIELD Antifade Mounting Medium containing DAPI (Vector Laboratories, Newark, CA, USA) by employing a ZEISS Apotome 40× magnifying lens (Carl Zeiss, Oberkochen, Germany).

### 4.5. Immunohistochemistry

Fetal human kidneys were obtained from medical abortions (20 and 23 weeks of gestational ages, respectively) and from a case of transient Bartter’s syndrome (22 weeks of gestational age) as described previously [3]. Kidneys were embedded with paraffin and cut into 5 μm thick sections. The sections, after being deparaffinized with xylene and graded alcohol, were rehydrated in a succession of 100%, 90%, 80%, 70% and 50%. Staining with rabbit LC3B (1:500) and mouse cytokeratin (1:100) antibodies in 1% bovine serum albumin (BSA) was performed at 4 °C overnight. Both Alexa Fluor Plus 555 and Alexa Fluor Plus 488 coupled secondary antibodies (1:500) were added the following day after three PBS washes and kept for 1 h at room temperature. Autofluorescence was removed by TrueVIEW Autofluorescence Quenching Kit (Vector Laboratories, Newark, CA, USA). Mounting with VECTASHIELD Antifade Mounting Medium containing DAPI (Vector Laboratories, Newark, CA, USA) preceded visualizing the sections using ZEISS Apotome (Carl Zeiss, Oberkochen, Germany).

### 4.6. Western Blotting

Lysis buffer (50 mM Tris pH 7.4, 5 mM EDTA, 150 mM NaCl, 1% Triton X-100 and protease inhibitors) was used to lyse the confluent cells after three ice-cold PBS washes. The lysates were centrifuged for 15 min at 13,000× *g*, and the protein concentrations of the supernatants were measured by a Pierce™ BCA Protein Assay Kit (Thermo Scientific™, Dreieich, Germany). We used 12% TGX gels Stain Free gels (Bio-rad, Feldkirchen, Germany; Cat. 1610181) to separate the proteins before blotting them to nitrocellulose membranes 0.2 µM through a Trans-Blot Turbo Transfer System (Bio-rad, Feldkirchen, Germany). Identifying the proteins was carried out by fluorescence antibodies StarBright Blue 520 and 700 (Bio-rad, Feldkirchen, Germany) using the ChemiDoc MP imaging machine (Bio-Rad, Feldkirchen, Germany). ImageJ software provided by the National Institutes of Health, Bethesda, MD, USA, was used to assess the gray density of Western blots. The signal for the protein of interest is normalized to the total amount of protein loaded into the lane using a stain-free membrane [47].

### 4.7. Quantitative Real-Time Reverse Transcription Polymerase Chain Reaction (qRT-PCR)

The quantities of ATG5 and ATG12 mRNA were measured using quantitative real-time RT-PCR (qRT-PCR), with the GAPDH gene serving as an internal control. Cell total RNA was extracted using the Bio-rad SingleShot Cell Lysis Kit and transcribed to complementary DNA (cDNA) using the Bio-rad iScriptTM Advanced cDNA Synthesis Kit. PCR was performed employing a QuantStudio™ 3 Real-Time PCR System (Applied Biosystems, Waltham, MA, USA) with SsoAdvanced Universal SYBR Green Supermix (Bio-rad). Cycle threshold values were normalized to GAPDH amplification.

### 4.8. Statistical Analyses

Results are displayed using mean ± SEM. Unpaired Student’s *t*-tests were used to analyze mean differences. Statistical analyses were carried out with the aid of the program GraphPad Prism X9. *p* values of 0.05 or less were deemed statistically significant (*), *p* values of 0.01 or less were deemed highly significant (**), and *p* values of 0.001 or less were deemed extremely significant (***).

## Figures and Tables

**Table 1:** Reagents and tools.

Reagent or Resource	Source	Identifier
**Antibodies**		
Anti-MAGED2 rabbit raised against this peptide (QVQENQDTRPKVKAK)	Eurogentec (Cologne, Germany)	
Anti-LC3B	Thermo Fisher Scientific (Dreieich, Germany)	PA1-46286
Cytokeratin	Agilent	M0821
ATG5 Antibody	Santa Cruz Biotechnology	sc-133158
P62 Antibody	Santa Cruz Biotechnology	sc-28359
Goat anti-rabbit IgG (H + L), Alexa Fluor Plus 555	Thermo Fisher Scientific (Dreieich, Germany)	A32732
Goat anti-rabbit IgG (H + L), Alexa Fluor Plus 555	Thermo Fisher Scientific (Dreieich, Germany)	A32732
StarBright Blue 520 Goat Anti-Rabbit IgG	Bio-rad (Feldkirchen, Germany)	12005869
StarBright Blue 700 Goat Anti-Mouse IgG	Bio-rad (Feldkirchen, Germany)	12004158
**Chemical stressors**		
Cobalt(II) chloride hexahydrate	Sigma Aldrich (Schnelldorf, Germany)	C8661
Tunicamycin	Cayman Chemical (Ann Arbor, MI, USA)	11445
Camptothecin	Cayman Chemical (Ann Arbor, MI, USA)	11694
2-deoxy-D-glucose	TCI (Eschborn, Germany)	D0051
Brefeldin A	Sigma Aldrich (Schnelldorf, Germany)	B6542
**Transfection**		
DhramaFECT4	Dharmacon (Cambridge, UK)	T-2004-03
**Critical Commercial Assays**		
SingleShot Cell Lysis Kit	Bio-rad (Feldkirchen, Germany)	1725080
iScript Advanced cDNA Synthesis Kit for RT-qPCR	Bio-rad (Feldkirchen, Germany)	1725038
SsoAdvanced Universal SYBR Green Supermix	Bio-rad (Feldkirchen, Germany)	1725271
**Experimental Models: Cell Lines**		
HEK293	ATCC	CRL1573
HeLa	Gift from Dr. Vijay Renigunta	
U2OS	Gift from Prof. Thorsten Stiewe, ZIT, Philipps-University, Marburg	
**Oligonucleotides**		
ON-TARGETplus Non-targeting Control Pool	Dharmacon (Cambridge, UK)	D-001810-10-05
UGGUUUACAUGUCGACUAA		
UGGUUUACAUGUUGUGUGA		
UGGUUUACAUGUUUUCUGA		
UGGUUUACAUGUUUUCCUA		
ON-TARGETplus Human MAGED2 siRNA—SMARTpool	Dharmacon (Cambridge, UK)	L-017284-01-0005
GGACGAAGCUGAUAUCGGA		
GCUAAAGACCAGACGAAGA		
AGGCGAUGGAAGCGGAUUU		
GAAAAGGACAGUAGCUCGA		
ON-TARGETplus Human GNAS siRNA—SMARTpool	Dharmacon (Cambridge, UK)	L-010825-00-0005
GCAAGUGGAUCCAGUGCUU		
GCAUGCACCUUCGUCAGUA		
AUGAGGAUCCUGCAUGUUA		
CAACCAAAGUGCAGGACAU		
GAPD, Human GAPDH, Real-Time PCR Primer Set	Biomol (Hamburg, Germany)	VHPS-3541
TTTTGGCTAAAGACCAGACG		
AATAGCCTGCTCGTTCAATG		
ATG5	Sigma-Aldric (Schnelldorf, Germany)	
AAAGATGTGCTTCGAGATGTGT		
CACTTTGTCAGTTACCAACGTCA		
ATG12	Sigma-Aldrich (Schnelldorf, Germany)	
CTGCTGGCGACACCAAGAAA		
CGTGTTCGCTCTACTGCCC		
**Software and Algorithms**		
ImageJ	Schneider et al., 2012 [46]	https://imagej.nih.gov/ij/, accessed on 22 July 2022
GraphPad Prism 9	GraphPad	
EndNote X9	Clarivate Analytics	
BioRender		https://www.biorender.com/, accessed on 22 August 2023

## Data Availability

All data are available in the main text or the Supplementary Materials.

## Supplementary Materials

The supporting information can be downloaded at: https://www.mdpi.com/article/10.3390/ijms241713433/s1.
