# Supplementary material for: MAGED2 Depletion Promotes Stress-Induced Autophagy by Impairing the cAMP/PKA Pathway
Source: Int J Mol Sci. 2023 Aug 30;24(17):13433. doi: 10.3390/ijms241713433 (PMC10488052; doi:10.3390/ijms241713433)
Supplement: Supplementary file 1 [file ijms-24-13433-s001.zip › ijms-2552358-supplementary.pdf]

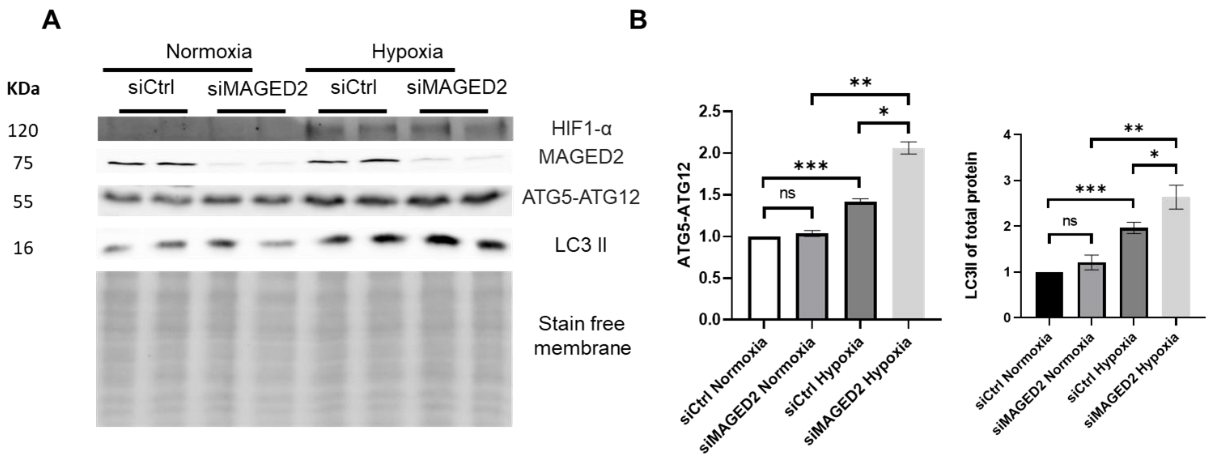

Figure S1. Compared to normoxia, autophagy is induced in HEK293 cells under hypoxic stress and MAGED2 silencing markedly enhances the autophagic induction under hypoxia.

Control or MAGED2 siRNAs were transfected into HEK293 cells. 24-48 hours post transfection, hypoxic stress was applied overnight for one set in a modular chamber (1% O_2, 5% CO_2, and 94% N_2) while the other set was kept in a humidified atmosphere (normoxia). Cells were then lysed and blotted for LC3B and autophagy related genes detection. Of note, HIF-1α immunoblotting confirmed the hypoxic condition (A) Representative western blot images from HEK293 cells reveal that hypoxia markedly induced autophagy in comparison to normoxia, as evident by upregulation of ATG5-ATG12 conjugate levels alongside the higher conversion to the lipidated form LC3II. MAGED2 depletion was dispensable under normoxic conditions in contrast to hypoxia where significantly augmented ATG5-ATG12 complex levels and increased LC3II abundance were observed. (B) Densitometric analysis of ATG5-ATG12 conjugate and LC3II from the immunoblot A. All samples shown on individual blots are from the same experiment and each blot represents an example of three independent experiments. Bar graphs show mean ±. SEM, * $p \leq 0.05$, ** $p \leq 0.01$, *** $p \leq 0.001$.

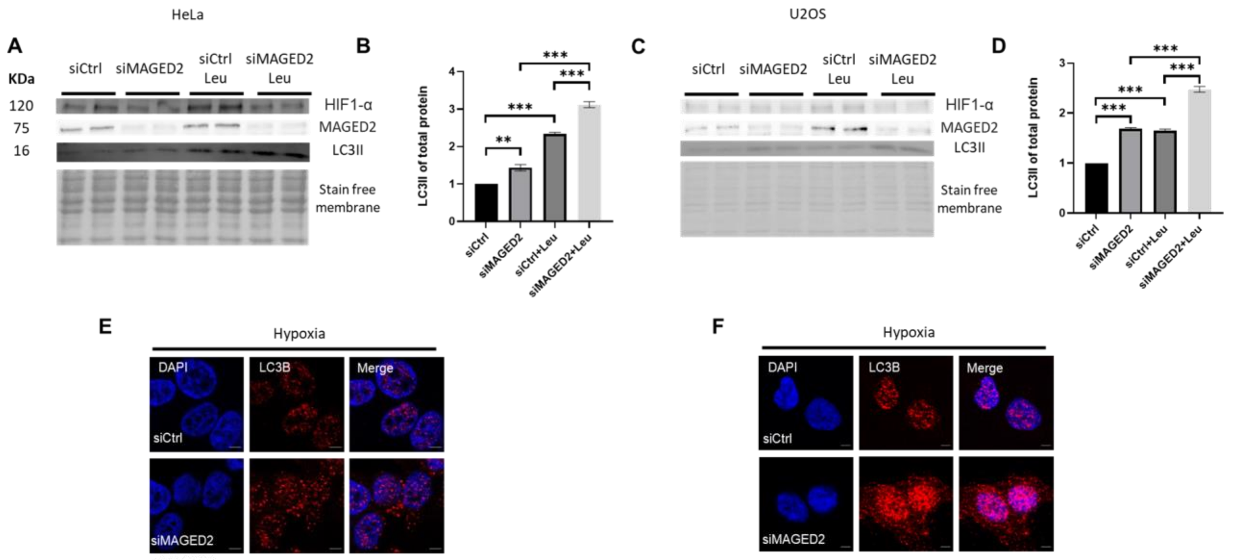

Figure S2. MAGED2 depletion induces autophagy under hypoxia in HeLa and p53-psoitive U2OS cells.

U2OS and HeLa cells were transfected with control and MAGED2 siRNA. 24-48 h post transfection, cells were exposed to physical hypoxia overnight. HIF-1α immunoblots confirmed hypoxia. A, C: Representative immunoblots disclose higher LC3II prevalence upon MAGED2 depletion in HeLa (A) and U2OS (C) cells. Treatment with leupeptin blocked the autophagic flux and resulted in the highest LC3II accumulation when MAGED2 is knocked-down. B, D: Densitometric analysis of LC3II in the immunoblots A, C respectively. All blots are from the same experiment and each represents an example of three independent experiments. Bar graphs show mean ± SEM, * $p \leq 0.05$, ** $p \leq 0.01$, *** $p \leq 0.001$. Immunocytochemistry in HeLa (E) and U2OS (F) cells, transfected with control or MAGED2 siRNAs and exposed to physical hypoxia, reveal marked accumulation of LC3B puncta upon MAGED2 depletion. The scale bar is 5 µM.

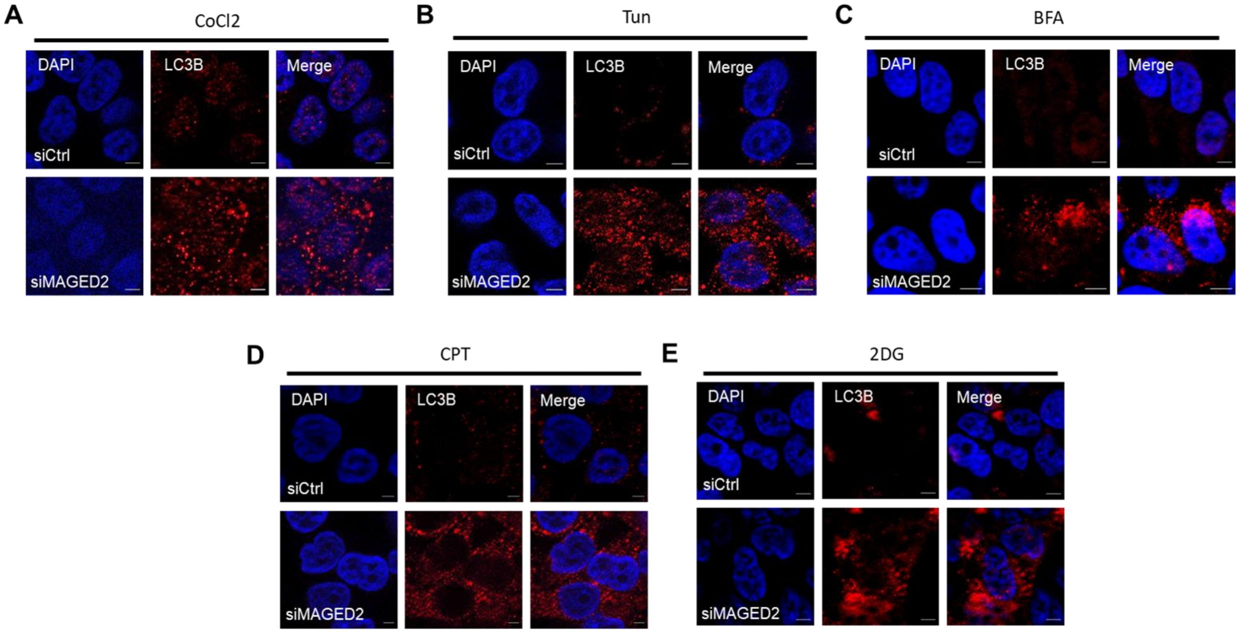

Figure S3. MAGED2 depletion in HeLa cells induces autophagy significantly in response to numerous stress conditions.

Control and MAGED2 siRNAs were transfected in HeLa cells. Upon confluency, 24-48 hours after transfection, cells were exposed to a variety of stress conditions; 300 µM $CoCl_2$ overnight, 600 nM Tunicamycin overnight, 10 µM Camptothecin overnight, 10 µM Brefeldin A for 2 hours and 4 mM 2-Deoxy-D-glucose for 30 minutes to avoid toxic effects. Immunocytochemistry was conducted to stain against LC3B in control and MAGED2 transfected HeLa cells which were treated with $CoCl_2$ (A), tunicamycin (B), BFA (C), CPT (D) or 2DG (E). The scale bar is 5µM.

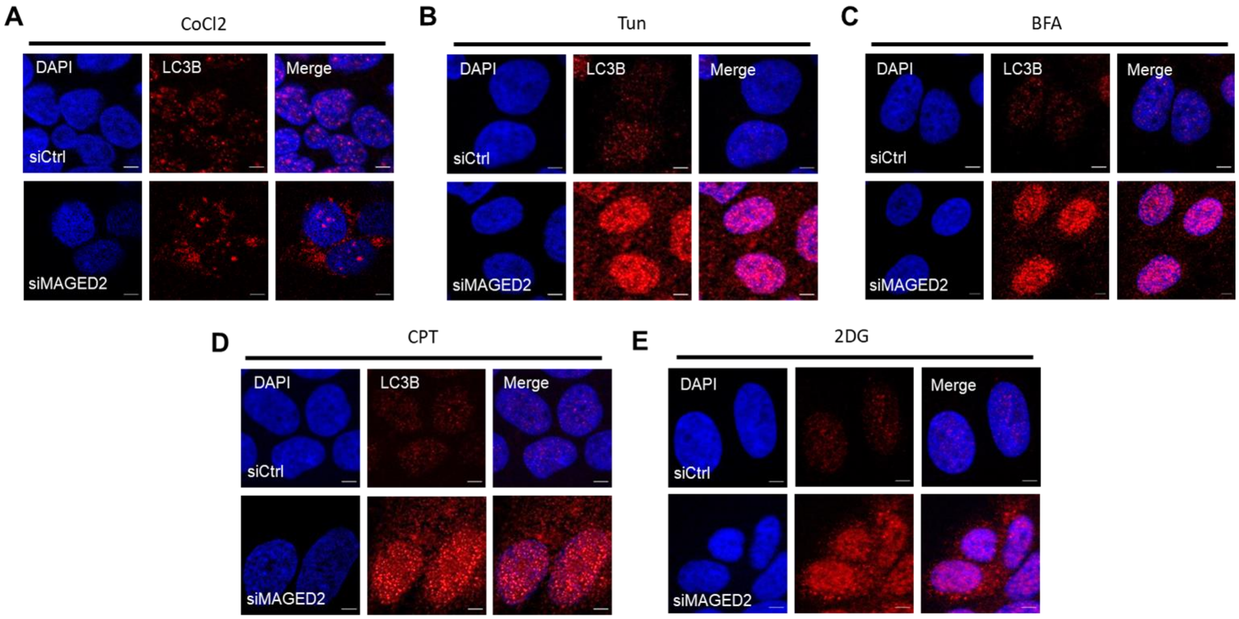

Figure S4. Stress-induced autophagy is significantly promoted in U2OS cells upon MAGED2 depletion.

U2OS cells were transfected with Control and MAGED2 siRNAs. 24-48 h post-transfection, cells were subjected to diverse stress conditions; 300 µM $CoCl_2$ overnight, 600 nM Tunicamycin overnight, 10 µM Camptothecin overnight, 10 µM Brefeldin A for 2 hours and 4 mM 2-Deoxy-D-glucose for 30 minutes to avoid toxic effects. Immunocytochemistry was performed to stain against LC3B in control and MAGED2 transfected U2OS cells which were treated with $CoCl_2$ (A), tunicamycin (B), BFA (C), CPT (D) or 2DG (E). The scale bar is 5µM.

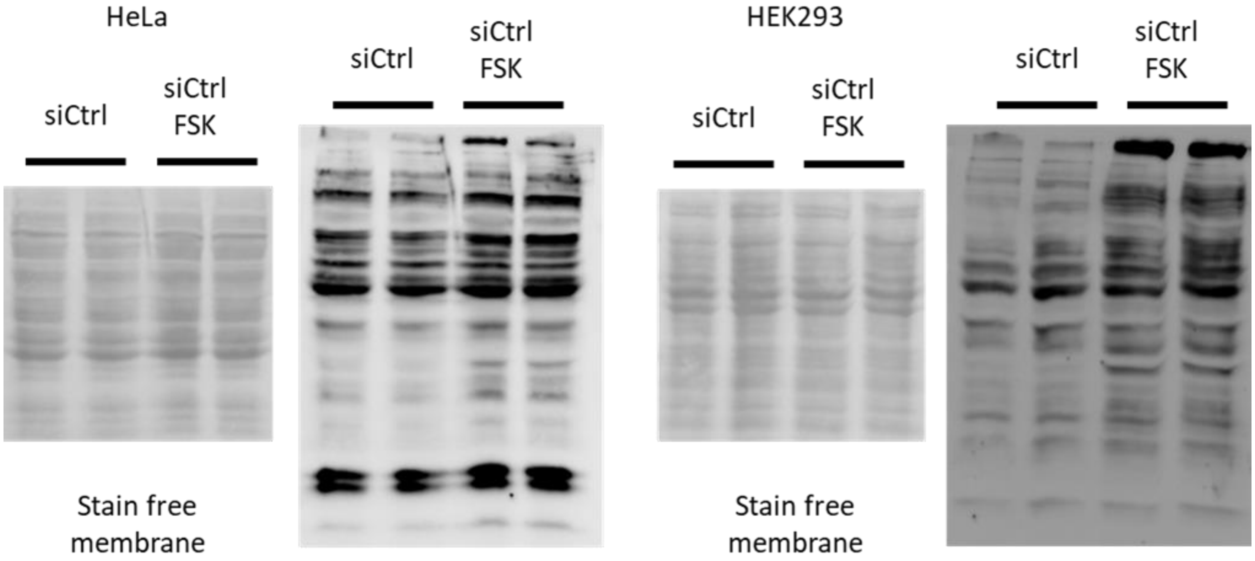

Figure S5. Forskolin treatment increases the phosphorylation of PKA substrates.

HeLa and HEK293 cells were transfected with control siRNA following our standard protocol and upon confluency 24-48 h post transfection, the medium of the cells was altered to either DMEM or DMEM containing 10 µM FSK. The cells were incubated overnight and the lysates were collected afterwards and blotted for phosphorylated PKA substrates.

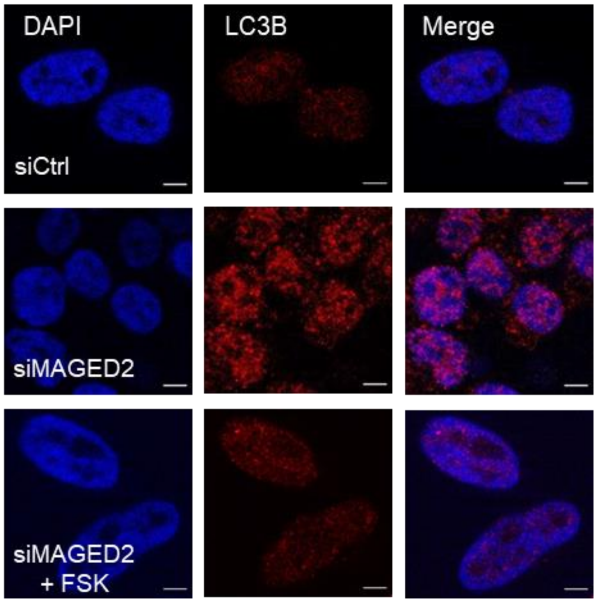

Figure S6. In MAGED2 depleted HeLa cells, forskolin treatment limits autophagy induction under ER stress.

HeLa cells were transfected with either control or MAGED2 siRNA. 24-48 h post transfection, the medium of the cells was changed to DMEM containing 600 nM tunicamycin or DMEM containing 10 µM forskolin together with 600 nM tunicamycin. The cells were stained for the accumulation of LC3B puncta and surprisingly the accumulation upon MAGED2 depletion was abrogated and rendered to control level when forskolin was added to the media.
